# Noncoding RNAs link metabolic reprogramming to immune microenvironment in cancers

**DOI:** 10.1186/s13045-021-01179-y

**Published:** 2021-10-15

**Authors:** Yiyin Zhang, Qijiang Mao, Qiming Xia, Jiaxi Cheng, Zhengze Huang, Yirun Li, Peng Chen, Jing Yang, Xiaoxiao Fan, Yuelong Liang, Hui Lin

**Affiliations:** 1 Department of General Surgery, Sir Run Run Shaw Hospital, School of Medicine, Zhejiang University, Hangzhou, 310016 China; 2 State Key Laboratory of Modern Optical Instrumentations, Centre for Optical and Electromagnetic Research, College of Optical Science and Engineering, International Research Center for Advanced Photonics, Zhejiang University, Hangzhou, 310058 China; 3 Zhejiang Engineering Research Center of Cognitive Healthcare, Sir Run Run Shaw Hospital, School of Medicine, Zhejiang University, Hangzhou, 310016 China

**Keywords:** Noncoding RNAs, Tumor metabolism, Immune microenvironment, TIMELnc manual, Bioinformatic approach

## Abstract

Altered metabolic patterns in tumor cells not only meet their own growth requirements but also shape an immunosuppressive microenvironment through multiple mechanisms. Noncoding RNAs constitute approximately 60% of the transcriptional output of human cells and have been shown to regulate numerous cellular processes under developmental and pathological conditions. Given their extensive action mechanisms based on motif recognition patterns, noncoding RNAs may serve as hinges bridging metabolic activity and immune responses. Indeed, recent studies have shown that microRNAs, long noncoding RNAs and circRNAs are widely involved in tumor metabolic rewiring, immune cell infiltration and function. Hence, we summarized existing knowledge of the role of noncoding RNAs in the remodeling of tumor metabolism and the immune microenvironment, and notably, we established the TIMELnc manual, which is a free and public manual for researchers to identify pivotal lncRNAs that are simultaneously correlated with tumor metabolism and immune cell infiltration based on a bioinformatic approach.

**Supplementary Information:**

The online version contains supplementary material available at 10.1186/s13045-021-01179-y.

## Background

When confronted with the severe nutritional crisis associated with increased interstitial pressure, destruction of vascular structures and hypoxia in the tumor microenvironment, tumor cells and other immunostromal cells experience conspicuous metabolic reprogramming [1–5]. In recent decades, many studies have deciphered alterations in metabolic profiles within tumor cells, and aberrantly activated metabolic pathways such as glycolysis and glutaminolysis allow tumor cells to sustain a higher proliferation rate and resist cell death signals [6–9]. However, altered metabolic patterns in tumor cells not only meet their own growth requirements but also shape an immunosuppressive microenvironment by disturbing the metabolism of other cells in the microenvironment through multiple mechanisms [2, 3]. Tumor-derived metabolites directly reduce the antitumor activity and recruitment of immune cells or indirectly compromise their function by inducing the formation of an acidic microenvironment [10–13]. Interestingly, tumor-derived metabolites were recently shown to enhance the function of suppressive immune cells, which dramatically restrained the cytotoxicity of antitumor immune cells [14]. In addition, reprogrammed metabolic pathways regulate the expression of immune checkpoints, while activated immune checkpoints in turn damage anticancer immunity by inducing metabolic reprogramming in T cells [15–17]. Hence, approaches concurrently targeting tumor metabolism serve as a synergetic strategy for immunotherapy.

However, recent studies implied that immunostromal cells also overcome these obstacles by triggering the metabolism-dependent death of tumor cells, which is a mechanism regulating the capability of tumors to plunder nutrients while bypassing the intratumor metabolite pool. For example, CD8+ T cells secrete IFN-γ to downregulate the expression of cystine/glutamate antiporter (SLC7A11) on the surface of tumor cells, which dramatically restrains the availability of cysteine, a key factor required for tumor cells to avoid lipid ROS accumulation-mediated cell death (ferroptosis) [18]. Moreover, because cysteine is an important nutritional substance for effector T cells to maintain their normal function, IFN-γ-mediated SLC7A11 downregulation might be a potent mechanism by which effector T cells hijack cysteine and improve their antitumor activity [19, 20]. Tumor cells were reported to tame multiple immunostromal cells to fuel their growth. Interestingly, metabolites derived from these tumor-educated cells also compromise antitumor immunity [21–23]. For instance, myeloid-derived suppressive cells (MDSCs) harness the glycolytic byproduct methylglyoxal to suppress effector T cell function and stimulate tumor development [24].

Nonetheless, the majority of previous studies focused on the direct effect of differentially expressed enzymes on metabolic rewiring and subsequent remodeling of the tumor microenvironment. However, with more awareness of the pathophysiological regulatory mechanism of noncoding RNAs [25–27], small molecules that regulate signaling pathways in cells or intercellularly through multiple pathways, such as binding to DNA, RNA and even proteins, researchers have gradually recognized the potential role of noncoding RNAs in bridging metabolism to anticancer immunity. Compared with the specific maps of interactions between metabolic enzymes and pathways, one noncoding RNA might simultaneously regulate different metabolic pathways via a competing endogenous RNA (ceRNA) network [28, 29], suggesting that some hub noncoding RNAs may exert essential functions at the crossroads of intratumoral metabolism and the immune microenvironment.

In fact, the role of ncRNAs, including miRNAs, lncRNAs and circRNAs, in tumor development has been reported by numerous studies [30, 31], and their effects on tumor metabolism have recently received increasing attention [32–36]. Overexpression of some ncRNAs counteracts the antitumor capability of effector T cells by triggering the aberrant upregulation of immunosuppressive metabolic activity [37, 38]. Stroma-derived metabolites induce an immunosuppressive environment through immune cell polarization and abnormal ncRNA expression to accelerate tumor development [10]. Immunosuppressive metabolic enzymes also destroy the antitumor function of cytotoxic immune cells by altering ncRNA expression [39]. Notably, ncRNAs play an important role in determining the metabolic activity of immune cells, their antitumor function and cell fate [40–42]. In this review, we mainly focused on the pivotal role of ncRNAs in the immunometabolic crosstalk between tumor cells and other cells in the tumor microenvironment, summarizing the current status and future perspectives in this field. Moreover, at the end of this review, we elaborated the Tumor Immuno-MEtabolic-LncRNA (TIMELnc) manual, which identifies potential lncRNAs regulating tumor metabolism and the immune microenvironment. Readers interested in this topic could refer to this manual to identify potential lncRNAs and design their experiments.

## Common paradigms for the crosstalk between intratumoral metabolic and immune activity

**Fig. 1:**
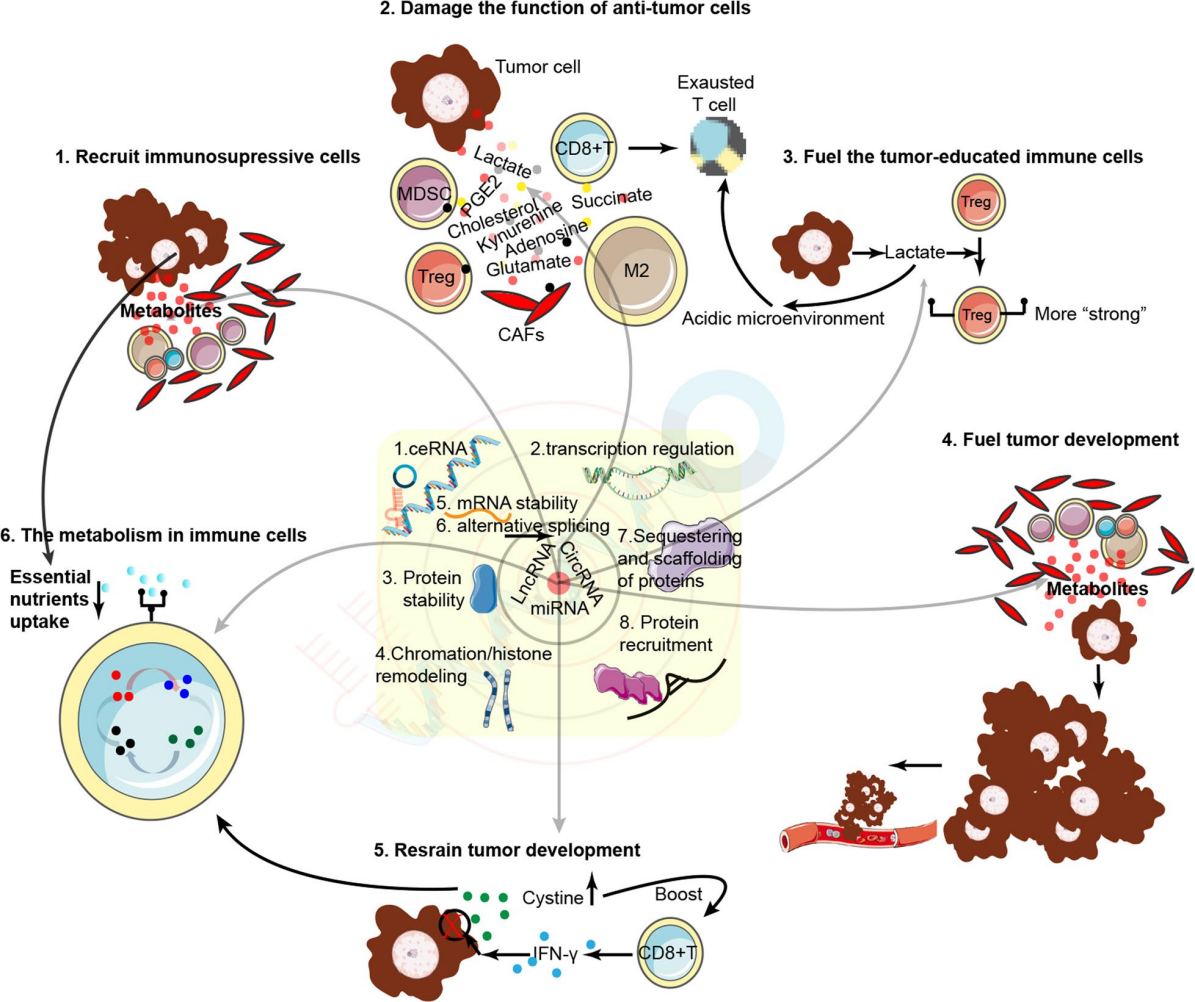
Noncoding RNAs regulate biological functions through multiple mechanisms, including ceRNA, transcriptional regulation, stabilizing/destabilizing proteins, chromatin/histone remodeling, stabilizing/destabilizing mRNAs, alternative splicing, sequestering and scaffolding of proteins and protein recruitment. Hence, given their polyfunctionality, noncoding RNAs may serve as hinges bridging metabolic activity and immune responses. Common patterns for the interaction of metabolism and the immune microenvironment were as follows: metabolites recruit or exclude immunosuppressive cells, damage or maintain the function of antitumor cells and fuel or restrain tumor-educated immune cells and tumor development

We summarized the common crosstalk patterns between intratumoral metabolism and immune activity in this section, which are potentially widely regulated by noncoding RNAs (Fig. 1).

### Tumor-derived metabolic products regulate the function of immune cells

#### Lactate

In the 1920s, Warburg and colleagues reported that tumor cells metabolize approximately ten-fold more glucose to lactate at a particular time than normal cells under aerobic conditions, which is known as the Warburg effect [43, 44]. In the next few decades, researchers focused on the benefits of the Warburg effect for tumor development, revealing that it not only supports the rapid proliferation of the tumor itself but also regulates the function of immune cells by producing a large amount of lactate [45, 46].

Excess lactate directly represses effector T cell and natural killer (NK) cell function and thereby establishes tumor immunosurveillance [47]. Mechanistically, lactate inhibits RLR signaling by directly binding to the MAVS transmembrane (TM) domain and preventing MAVS aggregation, which further impedes the production of IFN-γ by cytotoxic cells [13]. In addition, lactate upregulates the expression of PD-L1 on the tumor cell surface by activating the transcription factor TEAD and its coactivator TAZ [48]; then, elevated PD-L1 expression increases the number of exhausted T cells in the tumor microenvironment through its interaction with PD-1 [49, 50].

Nonetheless, tumor-derived lactate is not toxic to every cell type in the microenvironment. A recent study reported that lactate exposure enhances the function of Tregs. Lactate uptake is dispensable for the function of peripheral Treg cells but required within tumors. Blocking the uptake of lactate in Treg cells leads to slower tumor growth and an increased response to immunotherapy [14].

#### Glutamate

Most tumor cells consume glutamine at a high rate to sustain their rapid growth. Intriguingly, they simultaneously excrete glutamate, the first intermediate in glutamine metabolism. The reason why tumor cells are addicted to glutamine metabolism but upregulate glutamate excretion remains unclear. Nilsson et al. explained that glutamate excretion may help tumor cells increase the nucleotide synthesis rate to sustain growth [51]. However, based on accumulating evidence, tumor-derived glutamate facilitates tumor immune evasion. Glutamate exposure exerts a direct inhibitory effect on T cell proliferation and activation [52]. Excess accumulation of glutamate in the microenvironment restrains the uptake of cystine by antigen-activated T cells through the cystine-glutamate antiporter (xCT) and further dampens antitumor immunity [53]. Glutamine blockade not only abrogates the proliferation of tumor cells but also overcomes tumor immune evasion [54].

#### Kynurenine

Kynurenine, the first degradation product in the indoleamine 2,3-dioxygenase (IDO)-dependent tryptophan degradation pathway, has been reported to regulate immune cell function [55]. Kynurenine induces and activates aryl hydrocarbon receptor (AhR) and thereby upregulates PD-1 expression [56]. Moreover, an interaction between kynurenine and AhR generates more regulatory T cells by inducing naive T cell differentiation [57]. Furthermore, kynurenine depletion reverses IDO-mediated immune suppression and markedly increases the intratumor infiltration and proliferation of polyfunctional CD8+ lymphocytes [58].

#### Adenosine

The adenosinergic pathway is a major immunosuppressive mechanism and an attractive novel therapeutic target for cancer [15]. Meanwhile, extracellular adenosine serves as an essential immunosuppressive metabolite that restrains the maturation of NK cells and tumor-reactive effector T cells and then impairs antitumor immune responses [59, 60]. In contrast, inhibition of the adenosine receptor reduces the expression of T cell coinhibitory receptors and improves effector function for enhanced checkpoint blockade in preclinical cancer models [61]. In addition, the differentiation of regulatory T cells is obviously decreased upon inhibition of adenosine receptor [62]. The application of adenosine A_2A_ receptor antagonists for cancer immunotherapy in recent decades was summarized in a review [63].

#### Prostaglandin E2

As an essential homeostatic factor, prostaglandin E2 (PGE2) is also an important mediator of immunopathology in cancer. PGE2 directly impairs the function of NK cells through a mechanism involving the suppression of responsiveness to interleukins [64, 65] and indirectly restrains the NK cell function by abrogating the help from its adjuvant cells [66]. PGE2 also affects the induction of antigen-specific immune responses through the multifaceted regulation of DC functions to substantially reduce T cell-mediated immunity [67–69]. The inhibition of cytotoxic T lymphocytes mediated by PGE2 also contributes to tumor immune evasion [70, 71]. Moreover, PGE2 is also involved in the process of Ig class switching in activated B cells [72], Th cell polarization [73] and Th17 differentiation [74]. Notably, tumor-derived PGE2-mediated activation of nuclear p50 NF-κB epigenetically shifts the response of monocytic cells to IFN-γ toward an immunosuppressive phenotype, which enhances the anticancer properties of IFN-γ [75].

#### Other metabolites

In addition to the abovementioned classical immunoregulatory metabolites, recent studies also reported that some other tumor-derived metabolites potentially affect the function of immune cells. Fatty acids play an important role in the pathophysiological function of immune cells [17, 76–79]. Interestingly, recent studies showed that not all types of fatty acids exert the same function in antitumor immunity. Excess saturated fatty acids impair antigen presentation and NKT function by reducing CD1d expression on the cell surface, while polyunsaturated fatty acids decrease cancer progression by inducing an antitumor immune response [80]. Even as enantiomers, S-2-hydroxyglutarate treatment significantly increases the in vivo proliferation, persistence and antitumor activity of adoptively transferred CD8+ T cells [81], while tumor-derived R-2-hydroxyglutarate induces a perturbation in nuclear factor of activated T cell transcriptional activity and polyamine biosynthesis, leading to the suppression of T cell activity [82].

We summarized the reported metabolites involved in immune regulation in Table 1. In addition, their relationships with noncoding RNAs, which we will discuss in the following sections, are listed in Table 1.

**Table 1:** Immune regulatory metabolites and noncoding RNAs

Metabolites	References (PMID)	Role in antitumor immunity	Reported function in immune microenvironment	Noncoding RNAs participated in generation or utilization of the metabolite	References (PMID)
Acetate	31091446	Friend	Acetate promotes T cell effector function during glucose restriction	IFNG-AS1; miR-146a	33434756
Adenosine	29059149; 29229600	Foe	Activate antitumor immune responses and suppress natural killer cell maturation in the tumor microenvironment	NA	NA
Arginine	23017138; 27745970	Friend	Maintain T cell proliferation and proper function	miR-1291	33051382
Branched-chain amino acid	29141216; 27422517	Friend	Nanoliposome C6-ceramide increases the antitumor immune response and slows growth of liver tumors in mice	NA	NA
Ceramide	29408569	Friend	Nanoliposome C6-ceramide increases the antitumor immune response and slows growth of liver tumors in mice	miR-34a; miR-29b	32056304; 32746845
Cholesterol	32694690	Foe	Cholesterol-derived metabolites play complex roles in supporting cancer progression and suppressing immune responses	MeXis; MEG3; miR-128-1; miR-148a; miR-130b; miR-301b	29431742; 27770549; 26501192
Citrate	31751601	Friend	Citrate can impact the behaviors of both cancer and immune cells, resulting in induction of cancer cell apoptosis, boosting immune responses and enhanced cancer immunotherapy	NA	NA
Creatine	31628186	Friend	Creatine as an important metabolic regulator controlling antitumor T cell immunity, underscoring the potential of creatine supplementation to improve T cell-based cancer immunotherapies	NA	NA
Cysteine	20070126	Friend	Cysteine has a positive signaling effect (induction) on T cells proliferation and activation	LINC00618; miR-375; miR-139-5p	33002417; 28627030; 32109492
Lipid peroxidation byproducts	26073941; 33627871	Foe	Reverse the antitumor function by dendritic cells	NEAT1; circACC1; MACC1-AS1; LNMICC	29764424; 31155494; 30742067; 29229603
Glucose	23746840	Friend	Glucose-glycolysis-posttranslational modification of IFN-y mRNA-T cell effector function	miR-101 and miR-26a	26523864
Glutamate	25351939	Foe	Glutamate in turn has a direct inhibitory effect to T cell proliferation and activation	XLOC_006390; HOXA; Glu; CCAT2	31734356; 29844833; 32030797; 28934601
Glutamine	22885179	Friend	Maintaining optimal glutamine levels is critical in preventing the MDSC-mediated immunosuppression	LincRNA-p21; miR-137; miR-133a-3p; circHMGCS1; circHECTD1; GLS-AS; OIP5-AS1; lincRNA-p21; EPB41L4A-AS1; HOTAIR; UCA1; HOTTIP; TUG1; miR-122; miR-140-5p; miR-203; miR-23a/b; miR-513c; miR-153; miR-105; miR-9; miR-450a; miR-145; miR-103a-3p	30902882; 29348676; 30572959; 30809316; 31371702; 30563888; 30779126; 30902882; 30796006; 28597996; 22634383; 28800318; 28218035; 29662176; 29319172; 26373319; 26710269; 29371936; 32180557; 31986891; 30572959; 30035324; 31101765; 31115975; 31606381
Hyaluronic acid oligomers	30930171	Foe	Cancer cells secrete hyaluronic acid oligomers, thereby increasing cholesterol efflux in TAMs and directing TAMs toward an M2-like phenotype that accelerates tumor progression	NA	NA
Itaconic acid	29920191	Foe	Tumor-macrophagy-itaconic acid-tumor growth	miR93	28356443
Kynurenine	31068703; 29533786	Foe	The metabolite of Tryptophan that inhibits T effector activation and increases PD-1 expression	miR-153; ITGB2-AS1; HCP5; miR155HG; miR-30b	32165090; 29685162; 31093946
Lactate	31155231	Foe	Lactate is responsible for glycolysis-mediated RLR signaling inhibition to shrink from cancer immune surveillance	GLCC1; MALAT1; HULC; circDENND4C; miR-30a-5p; miR-21; Ftx; HOTAIR	31375671; 31953613; 32572027; 31488193; 28461244; 30664688; 29845188; 32062551
Microbiota/food-derived short-chain fatty acids	31272808	Friend	Microbiota-derived short-chain fatty acids promote the memory potential of antigen-activated CD8(+) T Cells	miR-106b; miR-125a; miR-30c; miR-182; miR-200c; let-7a	21283757; 31896754
Polyunsaturated fatty acids	30073695	Friend	Decrease cancer progression with an antitumor immune response	miR-138-5p	31783879
Prostaglandin E2	22187483	Foe	Enhance local accumulation of regulatory T cells and myeloid-derived suppressor cells and suppresses DC's ability to attract naive, memory and effector T cells	miR675-5p; miRNA-206; miR-21; miR-708-5p	31734182; 30135139; 29687845; 32655834
Pyrimidine	30827862	Foe	Macrophagy-pyrimidine-tumor-drug sensitivity	miRNA-375-3p	32073706
R-2-hydroxyglutarate	29988124	Foe	Tumor cell-derived R-2-HG is taken up by T cells where it induces a perturbation of nuclear factor of activated T cells transcriptional activity and polyamine biosynthesis, resulting in suppression of T cell activity	circRNA-51217	31846689
Retinoic acid	32169218	Foe	Tumor-derived retinoic acid regulates intratumoral monocyte differentiation to promote immune suppression	miR-124-3p; miR-302b; miR-29b; miR-664a-5p; miR-10a/b;miR-21	25753094; 25040912; 29619741; 29341475; 21131358; 21212796; 25934412; 21131358
S-2-hydroxyglutarate	27798602	Friend	S-2-hydroxyglutarate treatment greatly enhances the in vivo proliferation, persistence and antitumor capacity of adoptively transferred CD8+ T cells	NA	NA
Sarcosine	31753028	Friend	Sarcosine promotes trafficking of dendritic cells and improves efficacy of antitumor dendritic cell vaccines via CXC chemokine family signaling	NA	NA
Saturated fatty acids	31620124	Foe	Impair antigen presentation and NKT function by reducing the CD1d expression on surface	NA	NA
Succinate	33023985	Foe	Breast cancer-associated macrophages promote tumorigenesis by suppressing succinate dehydrogenase in tumor cells	miRNA-4470	31304868
Taurine	29945116	Friend	Alter splenocytes immunological profile of CD3+ CD4+ , CD3+ CD8+ , CD4+ CD25+ and CD11b+ Ly6G+ cells to achieve better immune surveillance against tumor cells	TUG1	30912122
Tetrahydrobiopterin (BH4)	30405245	Friend	Enhance antitumor immunity by promoting T cell activation and proliferation	miR-124; miR-206	31210282; 29436714
Triglycerides	20622859	Foe	Lipid accumulation and dendritic cell dysfunction in cancer	miR-132; HULC; NEAT1; miR-124-3p	25592151; 28381526
Tryptophan	23090118	Friend	Activate T cells; The target that IDO-expressing cells used to inhibit T cell activation	miR-448; miR-153; circZNF566; miR-18a; miR-669b-3p	31391111; 29685162; 32532962; 30268986; 32687860
Vitamin D	26811638	Friend	Vitamin D regulates immune cell trafficking and differentiation	CCAT2; MEG3; miR-155	32230936; 32219064; 30639520
22-hydroxycholesterol	23897983	Foe	Recruit neutrophils via oxysterol-CXCR2 axis	NA	NA
25-hydroxycholesterol	23541792	Foe	Interacts with G-protein-coupled receptor 183 and triggers migration of both macrophages and human blood monocytes by reorganizing the cytoskeletal protein vimentin8	miR-139-5p; miR-33a	28257846; 32109243
1-pyrroline-5-carboxylate	30545412	Foe	Attenuate T cell-mediated antitumor immunity	NA	NA
27-hydroxycholesterol	29021522	Foe	The pro-metastatic actions of 27-hydroxycholesterol requires both polymorphonuclear-neutrophils and γδ-T cells, and 27-hydroxycholesterol treatment results in a decreased number of cytotoxic CD8+ T lymphocytes	miR-933	29966198

### The metabolism of tumor-educated cells regulates the function of immune cells

Cancer-associated fibroblasts (CAFs) are some of the most critical components of the tumor stroma and not only provide physical support for tumor cells but are also key functional regulators of the tumor microenvironment. According to a recent study, CAFs reduce the percentage of the antitumor Th1 subset through lactate-dependent, SIRT1-mediated deacetylation/degradation of the T-bet transcription factor [10]. In addition, CAF exposure also increases the level of infiltrating Treg cells by driving naive T cell polarization through a mechanism dependent on lactate-mediated NF-kB activation and FoxP3 expression [10].

MDSCs decrease the availability of metabolites critical for T cell functions through multiple pathways. For example, MDSCs deplete L-arginine through four different enzymes, including nitric oxide synthases (NOS1-3), arginases (ARG-1 and ARG–2), arginine-glycine amidinotransferase and L-arginine decarboxylase [83]. In addition, MDSCs also increase the uptake of L-arginine from the tumor microenvironment by the CAT-2B transporter [84].

### The metabolism of tumor-educated cells fuels the progression of tumor cells

Many studies have reported that the metabolic activity of tumor-educated cells supports the proliferation and metastasis of tumor cells. As shown in the recent study by Sun et al., CAF-derived lactate promotes tumor cell progression by activating the TGFβ1 signaling pathways and enhances mitochondrial activity in tumor cells [85]. Tumor cells also take up lactate secreted by CAFs to fuel the TCA cycle, accumulation of oncometabolites and subsequent hypoxia-mediated EMT [86]. In addition, macrophage-derived succinate is likely a significant oncometabolite that induces tumor development by activating the TCA cycle [87]. Cancer cells can educate stromal cells to enhance their ability to use different nutrient sources for glutamine synthesis, which then supports tumor cell mitochondrial activity and de novo purine biosynthesis through glutaminolysis [88–90].

### Tumor metabolism regulates the recruitment of immune cells and remodels the physicochemical properties of the microenvironment

The metabolic activity of tumor cells has been reported to modulate gene transcription through multiple mechanisms, such as epigenetic modification [91–93]. Hence, a plausible speculation is that altered tumor metabolism disturbs the expression of some molecules involved in immune cell recruitment. Li et al. reported that glycolysis restriction inhibits the expression of granulocyte colony-stimulating factor (G-CSF) and granulocyte macrophage colony-stimulating factor (GM-CSF), which are essential chemotaxis molecules that recruit MDSCs [94]. Mechanistically, glycolysis inhibition restrains the expression of liver-enriched activator protein (LAP) through the AMP-activated protein kinase (AMPK)-ULK1 and autophagy pathways, whereas LAP controls G-CSF and GM-CSF expression to support MDSC development.

### The essential metabolic activities for immune cells to maintain normal function

Low tryptophan levels lead to cell cycle arrest and T lymphocyte apoptosis by activating the general control nonderepressible (GCN)-2 kinase [95]. The accumulation of tryptophan metabolites in the micromolar range in tumors leads to the differentiation of CD4+ T cells into a regulatory phenotype by binding to the aryl hydrocarbon receptor (AHR) and a reduction in T cell cytotoxicity [57]. In addition, lactate dehydrogenase inhibition promotes CD8+ T cell stemness and antitumor immunity [96].

STAT3 activation-induced fatty acid oxidation in CD8+ T effector cells is critical for obesity-induced breast tumor growth [97]. In contrast, tumor-infiltrating MDSCs tend to increase fatty acid uptake and activate fatty acid oxidation (FAO). Pharmacological inhibition of FAO blocks immune inhibitory pathways and the functions of these immunosuppressive cells to decrease their production of inhibitory cytokines. FAO inhibition alone significantly delays tumor growth in a T cell-dependent manner and enhances the antitumor effect of immunotherapy [98].

### Immunostromal cells regulate the metabolism in tumor cells

Breast cancer-associated macrophages promote tumorigenesis by suppressing succinate dehydrogenase activity in tumor cells. The decrease in SDH levels in tumor cells results in the accumulation of succinate, which increases the stability of the transcription factor HIF1α and reprograms cell metabolism to a glycolytic state [99]. In turn, HIF1α and glycolysis activation contribute to PD-L1 expression and failure of immunosurveillance, as previously observed in other cells in the tumor microenvironment [99, 100]. Tumor-associated macrophages (TAMs) secrete TNFα to promote tumor cell glycolysis, whereas depletion of TAMs by clodronate is sufficient to abrogate aerobic glycolysis [101]. TAM depletion leads to a significant increase in PD-L1 expression in aerobic cancer cells.

## The regulatory pattern and mechanism of noncoding RNAs in tumor biology

### The concept of noncoding RNAs

Noncoding RNAs constitute approximately 60% of the transcriptional output of human cells and have been shown to regulate numerous cellular processes and pathways under developmental and pathological conditions [25]. Based on the threshold of 200 nucleotides for RNA length, noncoding RNAs have been divided into lncRNAs and small RNAs [102], and the latter are further classified into several distinct RNAs, such as miRNAs, small nucleolar RNAs (snoRNAs) and piwiRNAs (piRNAs) [103]. Noncoding RNAs exert their biological functions through multiple mechanisms that bypass translation, such as inducing instability of target mRNAs [104], competitive endogenous networks [29], interactions with proteins [105] and transcriptional regulation [106]. Although the majority of noncoding RNAs have little protein-coding potential, many studies recently reported that some noncoding RNAs, such as circRNAs and lncRNAs [107, 108], potentially exert their functions by encoding peptides and regulating cancer development [109].

### Regulatory patterns and mechanisms of noncoding RNAs

#### Competing Endogenous RNA

LncRNAs and circRNAs can function as endogenous miRNA sponges [110–114]. ceRNAs communicate with each other by competing to bind to common miRNAs, thereby dictating miRNA availability [29]. The complementarity between the seed region of the miRNA and the 3′ untranslated region of the target mRNA mediates the cleavage of the latter [115]. Hence, lncRNAs or circRNAs could rescue the expression of some genes that are essential for cancer development by binding to their target miRNAs. In recent decades, ceRNA mechanisms have been shown to play important roles in tumor biology [28, 31, 116]. Different ceRNA combinations regulate the initiation [117, 118], growth [119–121], progression [122–124], metastasis [125–127], chemoresistance [128–130], apoptosis [121, 131, 132], stemness [133–135], recurrence [136–138] and metabolism [139–141] in various tumors. Moreover, the ceRNA network also modulates the tumor microenvironment by regulating stromal formation [142, 143], angiogenesis [144–146] and immune cell infiltration or function [147–150].

#### Transcriptional regulation

Many noncoding RNAs have been reported to directly regulate gene transcription or indirectly affect transcription factors [29, 151–153]. Li et al. have reported a class of circRNAs associated with RNA polymerase II in human cells, which is called ElciRNAs. In the ElciRNAs, exons are circularized with introns 'retained' between exons. These epithelial circRNAs are predominantly localized in the nucleus, interact with U1 snRNP and promote the transcription of their parental genes [151]. Similarly, lncRNAs were also reported to regulate transcription by binding to histone-modifying complexes, DNA binding proteins and RNA polymerase II [154]. An increasing number of studies have also shown that miRNAs may mediate transcriptional gene activation or silencing, which implies that miRNAs may not exclusively function at the posttranscriptional level. For instance, miR-373 induces the transcription of both E-cadherin and cold-shock domain-containing protein 2 [155], and miR-205 induces the transcriptional activation of the tumor suppressor genes IL24 and IL32.

#### Protein stability

Many noncoding RNAs have been shown to regulate protein stability. For example, the lncRNA LINRIS blocks the K139 ubiquitination of IGF2BP2, an oncogenic RNA-binding protein, maintaining its stability through the autophagy–lysosome pathway [156]. Similarly, another lncRNA, NEAT1, also directly binds to the DDX5 protein and regulates its stability, which sequentially activates Wnt signaling and exerts oncogenic functions [157]. The circRNA CDR1as was also reported to stabilize the p53 protein by preventing its ubiquitination. CDR1as directly interacts with the p53 DBD domain and thus disrupts p53/MDM2 complex formation, which inhibits gliomagenesis [158].

#### Chromatin/histone remodeling

Liquid–liquid phase separation is the basis for the formation of membrane-less organelles in cells and is involved in many biological processes [159]. Recently, many studies have shown that phase separation participates in chromatin/histone remodeling, and noncoding RNAs may also play an important role in this process [160–163]. Daneshvar and colleagues reported that the lncRNA DIGIT is required for bromodomain and extraterminal domain protein BRD3 to form phase-separated condensates [164], which is important for regulating endoderm differentiation. In addition to liquid–liquid phase separation, the lncRNA Xist silences the transcription of one X chromosome during development in female mammals by directly interacting, recruiting and activating a series of proteins and further deacetylating histones to exclude Pol II from the X chromosome [165]. Another approach by which noncoding RNAs indirectly modulate chromatin or histones is via epigenetic regulators. For instance, the lncRNA GATA6-AS epigenetically regulates gene expression through an interaction with LOXL2-mediated changes in histone methylation [166]. Reciprocally, H3K27 acetylation induces the expression of the lncRNA colon cancer-associated transcript-1 (CCAT1), whose overexpression may induce tumorigenesis in many cancers [167].

#### mRNA stability

In addition to miRNAs, other noncoding RNAs also showed the ability to directly influence mRNA stability. Chen recently revealed that circNSUN2 is upregulated in colorectal cancer and promotes liver metastasis by stabilizing the HMGA2 mRNA [168]. Similarly, Wu et al. reported that the lncRNA THOR increases osteosarcoma cell stemness and migration by increasing SOX9 mRNA stability. Further experiments indicated that the lncRNA THOR directly binds to the middle region of the SOX9 3'UTR, thereby increasing SOX9 mRNA stability and expression [169].

#### Alternative splicing

Alternative splicing is tightly associated with the transcription of noncoding RNAs, particularly circRNAs [170]. However, noncoding RNAs may subsequently regulate the alternative splicing of other genes [171]. Some lncRNAs, such as NEAT1 and MALAT1, potentially interact with splicing factors. An intimate association was observed between them and SC35 SF-containing nuclear speckles in both human and mouse cells, suggesting their participation in mRNA splicing [172]. Their role was further confirmed by the results of an RNA FISH analysis. Serine/arginine-rich (SR) proteins are a conserved family of proteins that are mainly involved in splicing. In addition, a continuous phosphorylation or dephosphorylation cycle of SR proteins is required for proper premRNA splicing and the regulation of AS patterns. According to recent studies, one SR protein, SRp40, directly recognizes NEAT1, exhibiting a dynamic association throughout adipocyte differentiation. Then, an increased concentration of the phosphorylated SRp40 protein after release from NEAT1 was proposed to promote the splicing of the PPARγ2 mRNA [173].

#### Sequestering, scaffolding and recruitment of proteins

Sun et al. reported that the novel lncRNA GClnc1 functions as a modular scaffold to recruit key components of the histone methyltransferase complex. Then, many oncogenic genes, such as SOD2, are activated epigenetically and mediate tumorigenesis [174]. In addition, Jie et al. [175] identified a novel circRNA named circMRPS35, which is associated with the clinicopathological characteristics and prognosis of patients with gastric cancer. Mechanistically, circMRPS35 functions as a scaffold to recruit histone acetyltransferase KAT7 to the promoters of the FOXO1 and FOXO3a genes, which catalyzes the acetylation of H4K5 in their promoters. Notably, circMRPS35 directly binds to FOXO1/3a promoter regions, thereby inducing the transcription of FOXO1/3a and triggering subsequent expression of downstream oncogenic genes [175].

#### Protein coding

Although noncoding RNAs are generally recognized to lack a protein-coding capability, recent studies have gradually shown that some of these molecules encode peptides and regulate biological processes in cancers. Huang and colleagues discovered that the lncRNA HOXB-AS3 encodes a conserved 53-aa peptide that suppresses colon cancer growth itself instead of its parental lncRNA [176]. In addition, Zhang et al. documented that an endogenous circRNA generated from a lncRNA encodes a regulatory peptide using ribosome nascent-chain complex-bound RNA sequencing [177]. This peptide directly interacts with the polymerase-associated factor complex and inhibits the transcriptional elongation of multiple oncogenes, thereby fueling glioblastoma tumorigenesis. Notably, Pan et al. previously summarized three categories of noncoding RNA-encoded peptides: miRNA-encoded peptides, a 90 residue-long regulatory peptide encoded by an lncRNA, and a circRNA-encoded truncated NCX1 protein [178].

#### Other functions

Significant enrichment of miRNAs has been observed in the nucleolar region of cells [179]. Many studies have reported the potential biological functions of nucleolar miRNAs in biological processes [180, 181]. For example, nucleolar RNA was observed to be colocalized with 28S ribosomal RNA, suggesting that miRNAs may associate with ribosome subunits at an early stage of ribosome biogenesis [182].

## Noncoding RNAs regulate both tumor metabolism and the immune microenvironment

**Fig. 2:**
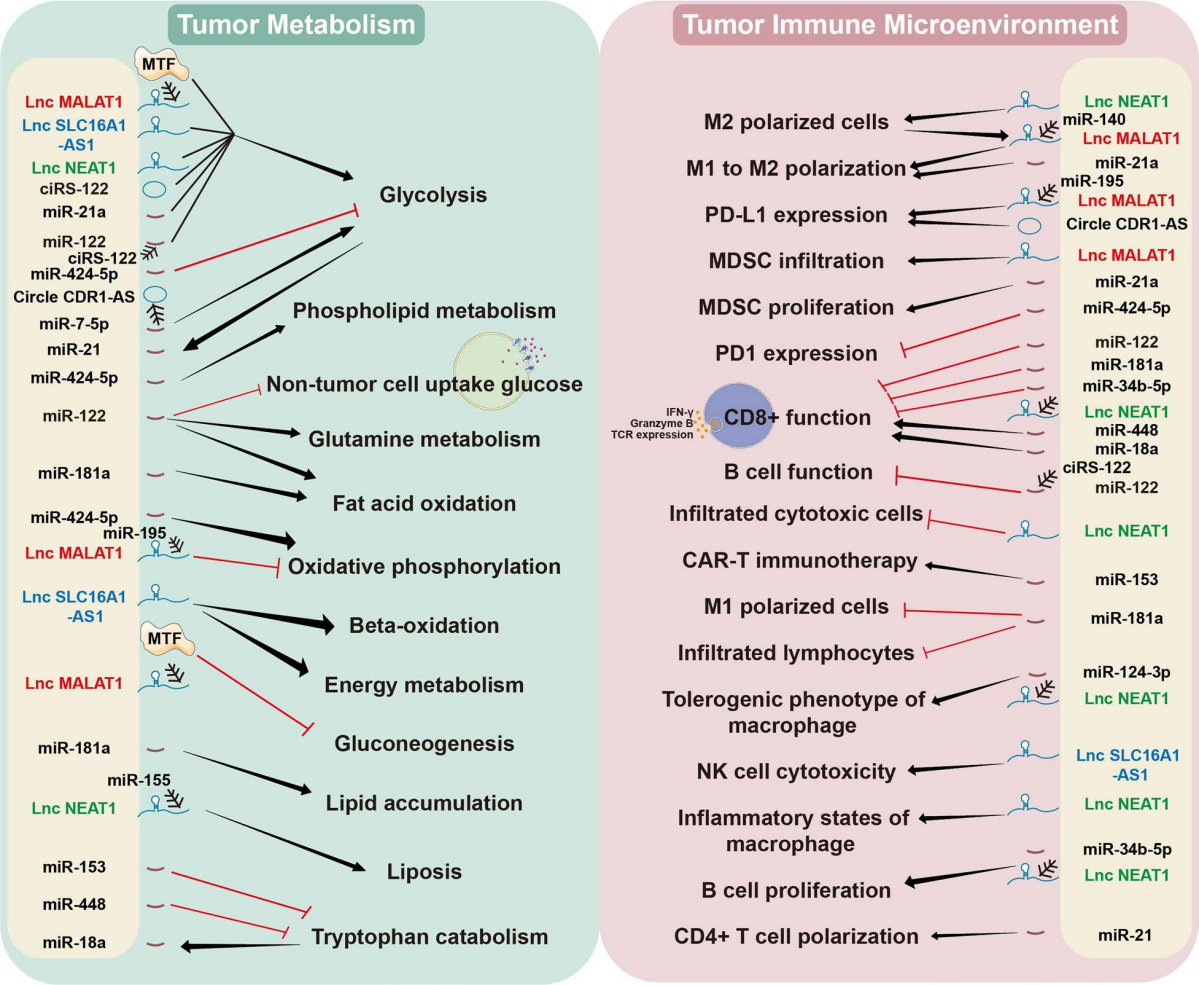
Existing knowledge has revealed the pivotal role of noncoding RNAs in bridging tumor metabolism and the immune microenvironment. (→ : promote;  : inhibit; the most frequently occurring noncoding RNAs have been marked in red, blue and green, respectively)

Since noncoding RNAs regulate many aspects of gene expression from pretranscriptional to posttranslational processes, as mentioned above, they are expected to exert effects on numerous cellular activities. In this context, miRNAs, lncRNAs, circRNAs and their regulatory networks may participate in the remodeling of tumor metabolism and the immune microenvironment (Fig. 2).

Tumor metabolism has always been a field studied by a wide range of researchers. Treatments targeting the addiction and dependence of tumor cells on reprogrammed metabolic pathways results in stringent tumor suppression in vivo and in vitro [9, 183, 184]. Many studies have reported that miRNAs, lncRNAs and circRNAs contribute to tumor metabolic rewiring, including glycometabolism [185], lipid metabolism [186] and amino acid metabolism [187]. Mechanistically, noncoding RNAs either directly regulate the mRNA and protein expression of metabolic enzymes or indirectly interact with the key factors that regulate the synthesis of metabolic enzymes.

Meanwhile, an increasing number of studies have emphasized the role of noncoding RNAs in remodeling the tumor microenvironment, which is involved in the immune cell differentiation trajectory, function and infiltration [188–194].

### Glycometabolism, noncoding RNAs and the immune microenvironment

Aerobic glycolysis or the Warburg effect is a representative hallmark of tumor metabolism. Through ceRNA mechanisms [139, 195–201], nucleolar translocation [202], protein interactions [156, 203, 204] and alternative splicing [205], noncoding RNAs reprogram glycolytic activity in tumor cells.

Interestingly, macrophage-derived lncRNAs were recently shown to regulate glycolysis in tumor cells. Extracellular vesicle-packaged HIF-1α-stabilizing lncRNA (HISLA) from tumor-associated macrophages regulates aerobic glycolysis in breast cancer cells by inhibiting the hydroxylation and degradation of HIF-1α. Reciprocally, the glycolytic product lactate upregulates the expression of HISLA in tumor-associated macrophages, which constitutes a feed-forward loop between TAMs and tumor cells [206]. Similarly, CAF-derived lactate modulates the polarization of CD4+ T cells by reducing the infiltration of Th1 cells and increasing the infiltration of Treg cells, a process in which miR-21 plays an important role [10].

In addition, Zhao et al. found that ovarian cancer cells imposed a glucose restriction on effector T cells and impaired their function by upregulating the expression of miR-101 and miR-26a, which constrained the expression of the methyltransferase EZH2 and the activity of the Notch pathway [207]. Consequently, the function of T cells was compromised due to the deficiency in Notch-mediated Bcl-2 signaling and polyfunctional cytokine expression [207]. Cancer-associated fibroblasts also mediate the upregulation of LINC00092, which further promotes glycolytic activity in ovarian cancer cells by stabilizing fructose-2,6-biphosphatase [208].

The lncRNA MALAT1 has been reported to regulate tumor cell metabolism through multiple mechanisms. For example, Malakar et al. reported that a metabolic transcription factor, TCF7L2, is stabilized by MALAT1 and mediates the upregulation of glycolytic activity but decreases gluconeogenic enzymes via the mTORC1-4EBP1 axis [209]. Similarly, Nanni et al. showed that MALAT1 silencing reduces the expression of some metabolic enzymes, including malic enzyme 3, pyruvate dehydrogenase kinases 1 and 3 and choline kinase A, which promotes a glycolytic phenotype and increased lactate production [210]. As we have described in the previous sections, the activation of glycolysis in tumor cells triggers an immunosuppressive tumor microenvironment. Hence, MALAT1 is expected to be a negative regulator of antitumor immunity. In fact, many studies have shown that MALAT1 exerts immunosuppressive effects in recent years. As shown in the study by Wang et al., MALAT1 sponges miR-195 to increase the expression of PD-L1, thereby promoting immune escape in diffuse large B cell lymphoma [147]. Likewise, through a ceRNA mechanism, MALAT1 overexpression contributes to angiogenesis and impairs M1 macrophage polarization by binding to miR-140 in hepatocellular carcinoma [211]. However, not all studies support the role of MALAT1 in promoting the immune evasion of tumors. Zhou et al. noted a negative correlation between the relative expression of MALAT1 and the proportion of MDSCs, while knockdown of MALAT1 significantly increases the proportion of MDSCs in the peripheral blood of patients with lung cancer [212].

Interestingly, immune cells facilitate tumorigenesis by inducing MALAT1 expression. For example, IL8 secreted from M2-polarized macrophages promotes prostate cancer progression via the STAT3/MALAT1 pathway, while knockdown of MALAT1 expression levels in prostate cancer cell lines inhibits cell proliferation, invasion and tumor formation [213].

As one of the most aberrantly expressed miRNAs detected in human cancers, miR-21a was reported to increase lactate generation and decrease oxygen consumption in lung cancer cells. Mechanistically, miR-21a directly targets fructose-1,6-biphosphatase and thereby reduces oxidative phosphorylation and increases glycolysis [214]. In this context, miR-21a may contribute to the formation of an immunosuppressive microenvironment by releasing glycolytic byproducts. Notably, miR-21a within MDSCs maintains the immunosuppressive microenvironment by enhancing the infiltration and function of polymorphonuclear MDSCs through the suppression of the MLL1 complex, while tumor-derived miR-21 also promotes the expansion of MDSCs by downregulating PDCD4 expression [215, 216]. Additionally, miR-21 promotes the transformation of macrophages toward M2 subtypes and further compromises antitumor immunity [217].

### Lipid metabolism, noncoding RNA and the immune microenvironment

According to a previous study, miR-424-5p modulates glucose metabolism during tumor growth and metastasis [218]. However, recent studies also showed that it affects lipid metabolism in cancer cells. Notably, miR-424-5p binds to ACSL4 and abrogates ferroptosis, a cell death mechanism dependent on lipid peroxidation, in ovarian cancer cells. In contrast, knockdown of miR-424-5p increases the sensitivity of ovarian cancer cells to ferroptosis inducers [219]. Moreover, miR-424-5p reduces the expression of phospholipid scramblase, which is responsible for collapsing lipid asymmetry by catalyzing bidirectional transbilayer movement of the major classes of phospholipids [220]. As a tumor suppressor, miR-424-5p also participates in regulating the expression of effector cytokines in T cells by promoting PD-L1 degradation [221]. The paradox is that miR-424-5p abrogates ferroptosis but promotes antitumor immunity; however, previous studies showed that ferroptosis potentially serves as an immunogenic form of cell death that triggers robust antitumor immunity [222, 223]. Future studies are expected to elucidate the mechanism underlying this contradiction. In addition, miR-122 controls fatty acid β oxidation by interacting with SIRT6 and predicts the prognosis of hepatocellular carcinoma [224]. Through immunoregulatory mechanisms, exosome-derived miR-122 drives tumor immune evasion by regulating TCR signaling and TNFα secretion [192].

As shown in previous studies, miR-181a is an essential modulator that regulates immune responses [192, 225], such as T cell differentiation [226, 227]. A recent study proposed that miR-181a also inhibits innate immune signaling by interrupting the STING-associated IFNγ response and lymphocyte infiltration in patients with cancer [228]. Moreover, Jiang et al. showed that exosome-derived miR-181a promotes the expansion of early-stage MDSCs by targeting PIAS3, a member of the protein inhibitor of activated STAT family, in breast cancer [229]. Chu et al. also showed that miR-181a decreases the expression of genes involved in lipid synthesis and increases the expression of genes involved in β-oxidation, subsequently inhibiting lipid accumulation in transgenic mouse models [230].

The long noncoding RNA NEAT1 has been reported to drive tumorigenesis and metastasis in multiple cancers [157, 231–234]. Liu et al. recently reported that lncRNA-NEAT1 modulates the expression of adipose triglyceride lipase (ATGL) and disrupts lipolysis in hepatocellular carcinoma cells [235]. In contrast, NEAT1 knockdown attenuates HCC cell growth through miR-124-3p and rescues lipolysis. NEAT also manifests an immunoregulatory function in cancers. Tumor samples with high levels of cytotoxic CD8+ infiltration express NEAT1 at lower levels. Furthermore, NEAT1 promotes tumor growth by inhibiting cytotoxic T cell-mediated immunity through a decrease in the expression of cyclic GMP-AMP synthase stimulator of interferon genes [236]. NEAT1 also induces the activation of the NLRP3 inflammasome in dendritic cells or macrophages to regulate their functions and phenotypes [237, 238]. In addition, NEAT1 sponges miR-214 to regulate M2 macrophage polarization by regulating B7-H3 in multiple myeloma, which further promotes tumor immune evasion [239].

### Amino acid metabolism, noncoding RNAs and the immune microenvironment

Tryptophan metabolism has long been recognized as an immunosuppressive mechanism in cancers [240–242]. Recent studies have shown that many noncoding RNAs regulate IDO-1 expression, which catalyzes the conversion of tryptophan into kynurenine and promotes immune evasion by activating T regulatory cells and MDSCs and suppressing the functions of effector T cells and natural killer cells [243]. According to Wang et al., IDO1 is overexpressed in colorectal tumors and is negatively associated with patient survival. Interestingly, IDO1 expression is reduced by miR-153, which targets the 3' untranslated region of IDO1 transcripts. Overexpression of miR-153 significantly inhibits tumor growth and enhances CAR T cell immunotherapy [38]. This finding was validated by another study showing that miR-153 decreases tryptophan catabolism and inhibits angiogenesis in bladder cancer [33]. In addition, Lou et al. screened miRNAs targeting IDO1 using a dual luciferase reporter assay. Their results showed that miR-448 significantly downregulates IDO1 protein expression and thereby suppresses the apoptosis of CD8+ T cells [37]. Notably, IDO1 also suppresses antitumor immunity through noncoding RNA-dependent mechanisms. For instance, IDO1 impairs NK cell cytotoxicity by promoting miR-18a expression, which is required for NKG2D/NKG2DL silencing [39].

Glutamine addiction has long been known as the main feature of tumor metabolic rewiring that fuels tumor growth [244, 245]. As we have summarized in the previous sections, glutamine metabolism is tightly associated with tumor immune evasion. The lncRNA HOTAIR was reported to upregulate chemokine (C–C motif) ligand 2 and promote the proliferation of macrophages and MDSCs in hepatocellular carcinoma [246]. A plausible speculation is that HOTAIR then mediates tumor immune evasion through MDSC recruitment. Interestingly, HOTAIR was also found to increase the expression level of glutaminase, which is essential for glutamine metabolism and subsequent oncogenic processes [247]. In this context, HOTAIR may mediate the immunosuppressive response by upregulating glutamine metabolism.

### The role of noncoding RNAs in the metabolism of immune cells

Given the potent functions of noncoding RNAs in multiple processes, a biologically plausible hypothesis is that noncoding RNAs in immune cells regulate many processes, such as metabolism and effector functions [248–250]. For instance, overexpression of miR-30c in macrophages promotes M1 macrophage differentiation and function by increasing glycolytic activity [251], while miR-143 inhibits glucose uptake and glycolysis by decreasing the expression of glucose transporter 1 in T cells to interrupt T cell differentiation [252].

Regarding lipid metabolism, miR-33 inhibits fatty acid oxidation in macrophages by decreasing the expression of retinoic acid-producing enzyme aldehyde dehydrogenase family 1 both in vitro and in a mouse model [253]. In addition, microRNA-150 expressed in macrophages also regulates pathological lipid trafficking [254].

Manually interrupting metabolism in immune cells has become a novel treatment modality in recent years that may function through noncoding RNAs. Sheng et al. blocked glycolysis in malignantly transformed macrophages and dendritic cells using 3-bromopyruvate (3-BrPA). They found that 3-BrPA significantly inhibited the proliferation of malignantly transformed macrophages and dendritic cells in a dose-dependent and time-dependent manner. Utilizing an online database and experimental data, they showed that 3-BrPA inhibits malignant progression via the miR-449a/MCT1 axis, which blocks lactate transport [255].

## Noncoding RNAs bridge metabolites and pro- or antitumor immunity

Metabolites are one of the most active elements that regulate multifaceted biological processes in the tumor microenvironment, serving as either nutrients producing energy or wastes whose accumulation interrupts normal cellular function [256–258]. Metabolites in the microenvironment modulate antitumor immunity or immune evasion. In this section, we describe currently reported metabolites that either enhance antitumor immunity or promote immune evasion in Table 1, and noncoding RNAs participating in the generation or utilization of these metabolites are also presented.

### Noncoding RNAs regulate the generation or utilization of metabolites that promote antitumor immunity

Metabolites enhance antitumor immunity through multiple mechanisms, one of the most important of which is providing essential nutritional support for tumor-killing immune cells. Arginine is important for effector T cells to maintain their antitumor activity. An increase in the L-arginine concentration triggers global metabolic rewiring, including a shift from aerobic glycolysis to oxidative phosphorylation in activated T cells, and promotes the generation of central memory-like cells. In vivo experiments further showed that increased arginine levels endowed a mouse model with higher antitumor activity and prolonged survival [259]. Interestingly, MDSCs with increased arginase I expression is part of an important mechanism that induces an immunosuppressive microenvironment by restricting the availability of arginine and restraining effector T cell function [260]. According to previous studies, miR-1291 targets the rate-limiting enzyme argininosuccinate synthase and reduces arginine synthesis [261]. miR-1291-5p sensitizes pancreatic carcinoma cells to arginine deprivation through the regulation of arginolysis [262]. In addition, external L-arginine also regulates the expression of many noncoding RNAs and triggers downstream biological changes [263–266].

T cell activation is initiated by the specific binding of the T cell receptor to an antigenic peptide presented by the major histocompatibility complex on the surface of an antigen-presenting cell (APC). Then, many ligations of costimulatory molecules on the surface of T cells are engaged and induce a downstream cascade of signaling events and pathways that regulate the clonal expansion and differentiation of naive T cells into effector T cells. These interactions were determined by investigating membrane physiology, which is maintained partially by the catabolic cleavage of sphingomyelin and the subsequent generation of ceramide [267, 268]. Some studies have reported that noncoding RNAs regulate the generation of ceramide or downstream of ceramide-mediated biological processes. For example, miR-34a causes ceramide accumulation [269], while ceramide inhibits the proangiogenic activity of multiple myeloma through miR-29b [270].

Another mechanism by which metabolites regulate antitumor immunity is epigenetic modification. For instance, acetate serves as an alternative energy source for both cancer cells and immune cells when glucose is restricted. Acetate rescues the effector function of CD8+ T cells by promoting histone acetylation and chromatin accessibility, thus facilitating IFN-γ gene transcription and cytokine production in an acetyl-CoA synthetase-dependent manner [271].

As we described in the previous sections, IDO-mediated tryptophan degradation was a major cause of effector T cell dysfunction in the tumor microenvironment. Hence, tryptophan itself is very important for the tumor-killing function of effector T cells. Many noncoding RNAs have been reported to affect tryptophan metabolism, enabling cells to reverse metabolism-mediated immune suppression [37–39, 272, 273]. Similarly, glucose is the basic nutrient required for the activation of various immune cells, especially effector T cells [274]. Zhao et al. reported a novel mechanism by which primary cancer imposes glucose restriction on T cells and affects antitumor immunity. They found that miR-101 and miR-26a were imperative factors mediating the effects of glucose deprivation on T cell polyfunctionality, while T cells were activated, and the abundance of miR-101 and miR-26a was rapidly reduced [207].

Tetrahydrobiopterin (BH4) is an important enzymatic cofactor required for the synthesis of dopamine, serotonin and nitric oxide [275]. Recently, Cronin et al. reported that BH4 controls antitumor immunity by increasing intratumoral expansion and function [276]. Administration of BH4 to animal models markedly reduces tumor growth and rescues the impaired antitumor immunity mediated by tryptophan-kynurenine metabolism [276]. Notably, BH4 metabolism could also be regulated by many noncoding RNAs [277, 278], suggesting that treatments targeting BH4 metabolism by modulating noncoding RNAs might be a novel modality for cancer immunotherapy.

Other small metabolites, such as vitamin D [279], taurine [280] and cysteine [281], also enhance antitumor immunity. Vitamin D regulates immune cell trafficking and differentiation, taurine alters the splenocyte immunological profile of CD3+ CD4+, CD3+ CD8+, CD4+ CD25+ and CD11b+ Ly6G+ cells to achieve better immune surveillance against tumor cells, and cysteine exerts a positive effect on T cell proliferation and activation. Noncoding RNAs play an important role in the production or transportation of these metabolites [282–285], reciprocally, they might also exert their functions through noncoding RNAs [286–288].

### Noncoding RNAs regulate the generation or utilization of metabolites that fuel tumor immune evasion

Cholesterol-derived metabolites play pivotal roles in supporting cancer progression and suppressing antitumor immune responses [289]. As shown in a recent study, tumor microenvironment-derived cholesterol increases CD36 expression and subsequent fatty acid uptake in tumor-infiltrating CD8+ T cells. Excess uptake of fatty acids triggers lipid peroxidation and ferroptosis in CD8+ T cells, which further leads to reduced cytotoxic cytokine production and impaired antitumor activity [290]. Even the hydroxylated products of cholesterol, 25-hydroxycholesterol or 27-hydroxycholesterol, induce immunosuppression by either promoting MDSC infiltration or decreasing CD8+ T cell numbers [291, 292]. Sallam et al. documented that the lncRNA MeXis promotes cholesterol efflux via the transcriptional regulation of the Abca1 gene [293], suggesting that MeXis may exert a similar function in tumor-associated macrophages to alter T cell function. In addition, Wagschal et al. leveraged a meta-analysis of genome-wide association studies and identified four microRNAs, including miR-128-1, miR-148a, miR-130b and miR-301b, involved in cholesterol-lipoprotein trafficking [294]. The metabolism of hydroxylated cholesterol is also regulated by or functions via other noncoding RNAs [295–297]. Targeting related noncoding RNAs may contribute to cholesterol-dependent immune suppression. Similar to cholesterol, lipid peroxidation byproducts and triglycerides fuel abnormal lipid accumulation in tumor-associated dendritic cells and reduces their ability to prime T cells [298, 299]. Moreover, many noncoding RNAs have been reported to regulate lipid peroxidation byproducts [235, 300–303].

Unlike lactate and kynurenine, two well-known immunosuppressive metabolites that regulate and are regulated by many noncoding RNAs in tumor biology, itaconic acid was recently identified as a macrophage-specific metabolite that promotes tumor progression. As the product of immune-responsive gene 1-mediated (IRG1) catabolism of mitochondrial cis-aconitate, itaconic acid in tumor-associated macrophages is upregulated by tumor cells and in turn alters tumor metabolism [304]. Repression of itaconic production significantly slows tumor development. Based on in vitro experiments, miR-93s also decrease itaconic acid production through an IRG1-mediated mechanism. Then, the decreased itaconic acid production mediated by miR93 further promotes and sustains M2-like polarization, even under M1-like polarizing conditions [305].

Chemotherapeutic drug resistance is a common problem faced by many patients with late-stage tumors. Many drugs are first metabolized and then exert their functions within cells, such as gemcitabine [306]. Recently, Halbrook et al. reported that macrophages in pancreatic cancer release a spectrum of pyrimidine species, which decrease gemcitabine efficacy through molecular competition at the level of drug uptake and metabolism [307]. Genetic or pharmacological depletion of tumor-associated macrophages in pancreatic cancer resensitized these tumors to gemcitabine [307]. miR-375-3p was found to be widely downregulated in human colorectal cancer cell lines and tissues and was associated with sensitivity to 5-fluorouracil. Mechanistically, miR-375-3p directly targets thymidylate synthase and is cotransported with 5-FU [308].

### The potential role of ncRNAs in metabolic remodeling during immune checkpoint therapy

Approaches targeting immune checkpoints such as PD1/PDL1 and CTLA4 have been a popular treatment modality for many cancers, including bladder cancer, melanoma, lung cancer and breast cancer [309–312]. Because the regulation of ncRNAs in tumor biology is multilayered and plastic, a plausible hypothesis is that ncRNA disturbances might determine the efficacy of ICIs. Many studies have shown that ncRNAs affect the expression levels of immune checkpoint genes. For example, miR155 increases PD-L1 expression in lymphoma cells, recruits CD8+ T cells through the PD-1/PD-L1 interaction and inhibits CD8+ T cells [313]. In contrast, miR-873 inhibits PD-L1 expression by directly binding to its 3'-untranslated region [314]. Through ceRNA mechanisms, lncRNAs and circRNAs also regulate checkpoint expression and immune evasion [150, 315]. In addition, ncRNAs seem to affect checkpoint trafficking. Hong et al. reported that circ-CPA4 promotes the secretion of PD-L1-containing exosomes and triggers immune evasion [133]. Interruption of the expression of some ncRNAs synergistically improves the efficacy of ICI treatment [316]. Notably, most of these ncRNAs have been reported to be involved in cell metabolism, including cholesterol efflux [317], glycolysis [318] and oxidative phosphorylation [319].

Interestingly, ncRNAs might also mediate side effects of ICI treatment. Xia et al. revealed that ICIs induce exosomal trafficking of miR-34a-5p from macrophages to cause cardiac injury in vivo [320]. However, few studies have focused on the direct alterations of ncRNA profiles during ICI treatment, which might become a hot field in the near future. Some questions must be answered by conducting appropriate studies. First, do ncRNAs mediate ICI resistance through metabolic rewiring? Second, do treatments targeting ncRNAs and metabolic reprogramming optimize the efficacy of ICIs, as the response rates to ICIs were unexpectedly low? Third, do specific ncRNAs exert pivotal effects on metabolic networks and dramatically reprogram the metabolic pattern to promote immune cell activation or inactivation in the antitumor microenvironment? Enriched high-throughput whole transcriptional sequencing data for samples from individuals treated with ICIs will be valuable to answer these questions.

## Construction of the TIMELnc manual by reviewing the transcriptomic data for 28 cancers in TCGA (The Cancer Genome Atlas)

**Fig. 3:**
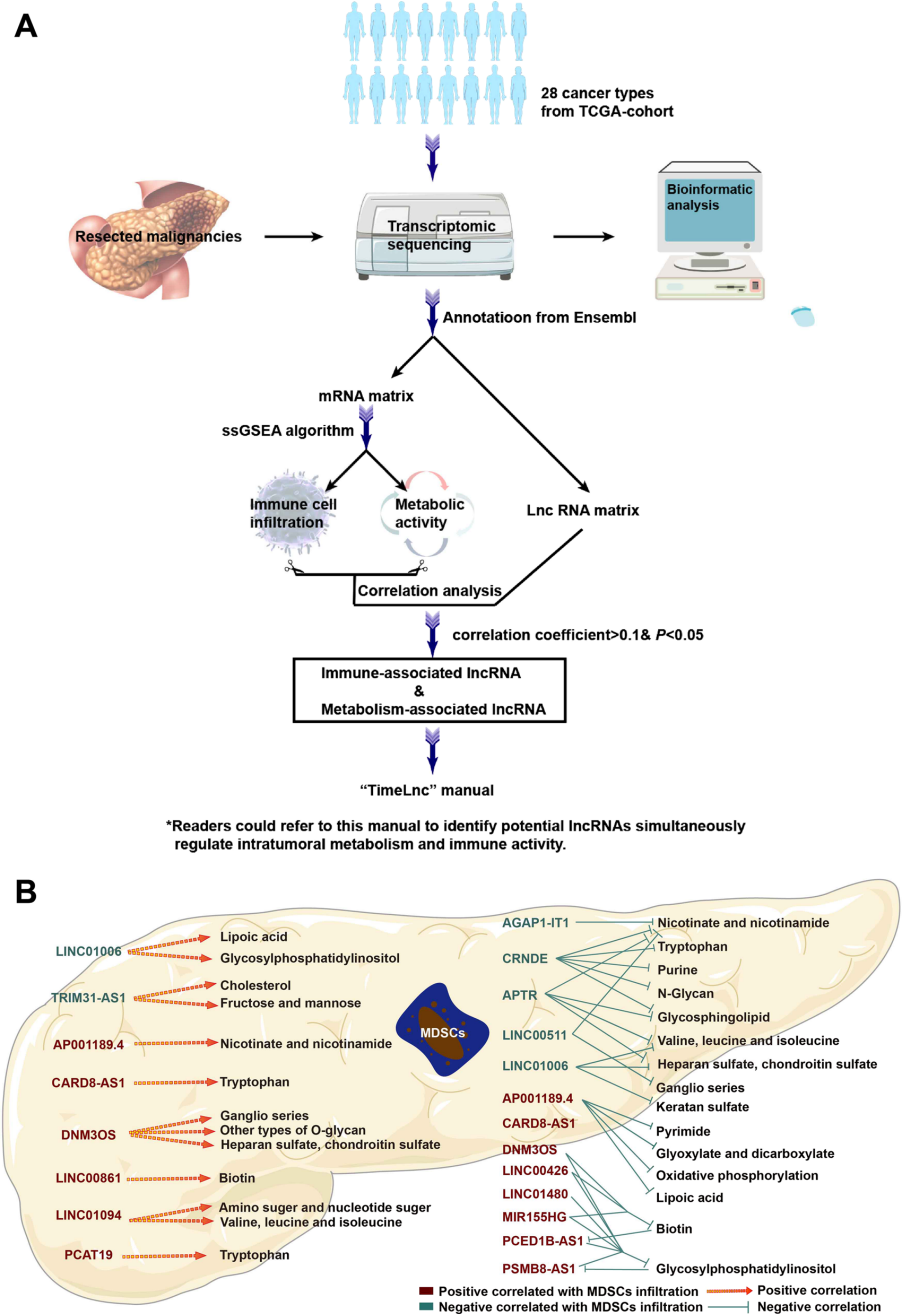
The TIMELnc manual was constructed to identify lncRNAs that are simultaneously correlated with tumor metabolism and immune cell infiltration. **a** Flow chart. **b** LncRNAs that are simultaneously associated with MDSCs and various metabolic pathways in pancreatic cancer are shown as an example

Despite the increasing number of studies reporting the roles of noncoding RNAs in tumor metabolism and the immune microenvironment, we speculated that many other noncoding RNAs involved in tumor immunity and metabolic reprogramming have yet to be identified given the broad regulatory mechanisms of noncoding RNAs in the physiopathology of tumors. LncRNAs play pivotal roles in the noncoding RNA regulatory network, and the intratumor expression of lncRNAs at the pancancer level can be accessed in public databases. In this context, we reanalyzed the transcriptomic data for 28 distinct cancers in TCGA database using the protocol shown in Fig. 3a and constructed the TIMELnc manual. This manual consisted of two sections: one section lists 85 lncRNAs associated with metabolic pathways, while the other section presents lncRNAs related to the infiltration of 28 immune cell types. Notably, we set an extremely low threshold (Pearson’s correlation coefficient (*r*) < 0.1) to screen potential lncRNAs of interest; hence, readers are recommended to set a higher threshold (e.g., *r* > 0.4) when using the manual if they want to select lncRNAs for further experimental validation.

We defined TIME-lncRNAs as those simultaneously correlated with intratumoral metabolic rewiring and immune cell infiltration. Readers could easily acquire mutually correlated metabolic pathway–lncRNA–immune signature pairs using the “screening” function in EXCEL. A representative screen of TIME-lncRNAs is described below. We set the threshold (*r* > 0.4) to screen metabolism-associated lncRNAs and immunity-associated lncRNAs in TCGA-PAAD cohort. An intersection was then acquired, and the metabolism–lncRNA–immunity network was established. We visualized the representative metabolism–lncRNA–MDSC correlation network in Fig. 3b. As shown in this figure, the lncRNA PCAT19 and CARD8-AS1 were positively associated with both tryptophan metabolism and MDSC infiltration, suggesting that their immunosuppressive role in the tumor microenvironment is potentially mediated by promoting immunosuppressive metabolism. Referring to the existing literature, PCAT19 is an oncogenic lncRNA that promotes tumor progression through multiple mechanisms [321–324], similar to CARD8-AS1 [325, 326]. In this context, their experimentally validated oncogenic functions paralleled their immunosuppressive roles we proposed using the TIMELnc manual, which also supported the value of applying TIMELnc in designing future studies. Readers can download the TIMELnc manual in the Additional files 1 and 2.

## Conclusions

Although more mechanisms underlying the intratumor interactions between metabolism and immune regulation have been deciphered in recent years, challenges and difficulties remain to be resolved before their effective “bedside” translation. One of the most important obstacles to metabolism-targeted treatment in cancers is that the activation of some so-called oncogenic pathways, such as anaerobic glycolysis, is also imperative for maintaining the antitumor function of effector immune cells [5, 327–329]. Under these circumstances, methods that precisely target metabolic pathways in tumor cells have become a bottleneck to the accelerated application of related regimens.

Our review summarizes existing knowledge of the role of noncoding RNAs in the remodeling of tumor metabolism and the immune microenvironment. Then, we proposed that noncoding RNAs potentially serve as hinges bridging metabolic activity and immune responses given their extensive action mechanisms based on motif recognition patterns. Target potential hub noncoding RNAs may simultaneously regulate multiple immunometabolic axes and reach optimal efficacy alone or in combination with immune checkpoint inhibitors. Hence, we also established the TIMELnc manual, which may help researchers screen these hub lncRNAs in future studies.

## Supplementary Information

**Additional file 1:** The TimeLnc manual in 28 cancers (Part 1: METABOLISM).

**Additional file 2:** The TimeLnc manual in 28 cancers (Part 2: IMMUNITY).

## Data Availability

The genelist of metabolic pathways was initially downloaded from KEGG (https://www.genome.jp/kegg/pathway.html), and the genelist to estimate immune cell infiltration in our study was based on a previously published study by Shao lab (PMID: 30837276).
